# Supplementary material for: Bisphosphonate nanoclay edge-site interactions facilitate hydrogel self-assembly and sustained growth factor localization
Source: Nat Commun. 2020 Mar 13;11:1365. doi: 10.1038/s41467-020-15152-9 (PMC7069965; doi:10.1038/s41467-020-15152-9)
Supplement: Supplementary file 1 — Supplementary Information [file 41467_2020_15152_MOESM1_ESM.pdf]

Supplementary Information

Bisphosphonate nanoclay edge-site interactions facilitate hydrogel self-assembly and sustained growth factor localization

Kim, et al.

Supplementary methods

Materials:

DL-dithiothreitol (DTT), 1-ethyl-3-(3-dimethylaminopropyl) carbodiimide (EDC), *N*-hydroxybenzotriazole (HOBt), *N*-Boc-ethylenediamine, tetrahydrofuran, triethylamine, acryloyl chloride, dichloromethane, dioxane, *N*-Boc-ethylenediamine were purchased from Sigma-Aldrich. Hyaluronic acid (HA) (150 kDa) was purchased from Lifecore Biomedical (US). Initiator Irgacure 2959 was purchased from BASF.

Preparation of HA derivatives:

Two types of HA-BP derivatives were used in which BP groups were linked either through disulfide bonds (HA-SS-BP) or by thiol-ene addition to HA-SH derivative yielding multiple BP ligand attachments (HA-BP_n).

The preparation of HA-SS-BP is shown in Supplementary figure 6 (a-b). Starting hyaluronan (HA) was firstly modified with 2-dithiopyridyl groups by EDC mediated amide coupling with 2-(2-pyridinyldithio)ethyl hydrazinecarboxylate linker) to give HA-SSPy (Supplementary figure 6a). Briefly, HA (400 mg, 1 mmol of disaccharide repeating units) was dissolved in 50 mL distilled water. 2-(2-pyridinyldithio)ethyl hydrazinecarboxylate linker (73.5 mg, 0.3 mmol) was dissolved in 4 mL of methanol and the solution was added into HA solution. A solution of HOBt (135 mg, 1 mmol) in 4 mL of acetonitrile:water (1:1 v/v) was subsequently added into the HA solution. pH of the reaction mixture was adjusted to 4.7. EDC (95.9 mg, 0.5 mmol) was added to the mixture

and the mixture was stirred overnight at room temperature. The mixture was dialyzed against acidified water (pH 3.5) containing 0.1M NaCl for 24 hours using dialysis membrane (MW cutoff = 3.5 kDa). After that, the dialysis medium was changed into acidified water (pH 3.5) twice and the dialyzed solution was lyophilized. Yield - 390 mg. The incorporation of 2-dithiopyridyl group was verified by ^{1}H-NMR. Specifically, the newly appeared peaks at 7.45, 7.92, 8.06, and 8.45 ppm corresponding to four aromatic protons of the pyridyl group were integrated and compared with the acetamido moiety of the *N*-acetyl-D-glucosamine residue of HA. It indicated that 25 % of HA disaccharide units were modified.

In the second step, HA-SS-BP was obtained by a thiol-disulfide exchange reaction between HA-SSPy and thiol-derivatized bisphosphonate (Supplementary figure 6b). HA-SSPy (200 mg, ≈0.125 mmol of –SSPy groups) was dissolved into 30 mL of de-ionized water. HA-SSPy solution (66.5 mg, 0.15 mmol) was mixed with dithiopyridyl:thiol at a molar ratio of 1:1.2 followed by adjusting pH of the mixture to 7.0. The reaction was performed at room temperature overnight. Thereafter, the mixture was transferred into a dialysis membrane with MW cutoff of 3.5 kDa and dialyzed against acidified water (pH 3.5) containing 0.1M NaCl once and then against acidified water (pH 3.5) twice. The resulting solution was finally lyophilized to obtain 185 mg of HA-BP. The structure of HA-BP was analyzed via ^{1}H-NMR (D_2O) and ^{31}P-NMR (D_2O).

The preparation of HA-SS-BP is shown in Supplementary figure 6 (c-d). HA (400 mg, 1 mmol of disaccharide repeating units) was dissolved in de-ionized water at concentration 8 mg/mL. Dihydrazide linker (Supplementary figure 6c) was added to the HA solution at the linker/HA disaccharide molar ratios 0.15 : 1. *N*-hydroxybenzotriazole (HOBt) was separately dissolved in a

1:1 (v/v) mixture of acetonitrile-water at concentration 0.2 M and added to the solution of HA. Molar ratio of HOBt to HA disaccharide was 1 : 1. The pH of the resultant solution was adjusted to 4.7 after which the coupling reaction was initiated by addition of solid EDC (0.15 molar equivalents per HA disaccharide units) to the reaction mixture. The mixture was stirred overnight and then basified to 8.5 with 1M NaOH. DTT was added to the solution. 10-fold molar excess of DTT relative to the estimated amount of disulfide linkages in the HA derivative was used to ensure the cleavage of disulfide bond by the reagent. The mixture was stirred overnight, after which the solution was transferred to a dialysis tube (M_w cutoff = 3500). After exhaustive dialysis against dilute HCl (pH 3.5) containing 0.1 M NaCl, followed by dialysis against dilute HCl, pH 3.5 two times. After lyophilization of the dialyzed solution, 369 mg of the thiol-modified HA (HA-SH) was obtained (92 % yield for the last step). The degree of incorporation of thiol (7 %) groups in HA-SH was verified by comparison of integration of the –C*H*2C*H*2SH side chain peaks at 2.58 and 2.73 ppm with the acetamido moiety of the *N*-acetyl-D-glucosamine residue of HA.

In the second step, HA-BP_n was obtained by a thiol-ene radical addition reaction between HA-SH and acrylamimde-derivatized bisphosphonate (Supplementary figure 6d). In brief, BP-acrylamide (24 mg, 0.2 mmol) was added to 100 mg of HA-SH in 12 mL degassed distilled water in order to obtain BP-to-thiol molar ratios of 4:1. Subsequently, 2.9 mg of radical initiator Irgacure® 2959 was added and the mixture was stirred for 10 min under ultraviolet light (36 W UV timer lamp, CNC international BV, Netherlands). Thereafter, the mixture was dialysed against 0.1 M NaCl at pH 3.5 (MW cutoff of 3.5 kDa) and subsequently dialysed (48 h) against distilled water at pH 3.5 twice. The solution was neutralised to pH 7.4 and lyophilised. Yield – 108 mg. The resulting polymers were analysed by ^{1}H NMR and ^{31}P NMR and elemental analysis (colorimetric spectrophotometric method by OEA Labs). Specifically, ^{1}H NMR peaks corresponding to the

native HA protons (such as acetamide protons at 1.9 ppm; 2′, 3′, 4′, 5′ and 6′-protons of the HA disaccharide unit between 3.2–4.0 ppm as well as anomeric 1′-protons at 4.4 ppm) were compared with peaks corresponding to the methylene protons 2 and 3 of the grafted side chains. The peak at 2.2 ppm corresponds to two methylene protons –C*H*$_2$C(OH)(PO_3H_2)$_2$ that are adjacent to a bridging carbon of the BP group. Assuming that bisphosphonate monomers can polymerize by addition of a thiyl radical, the elemental analysis data corresponds to approximately three BP-acrylamide molecules oligomerized from one thiol group.

Supplementary figures

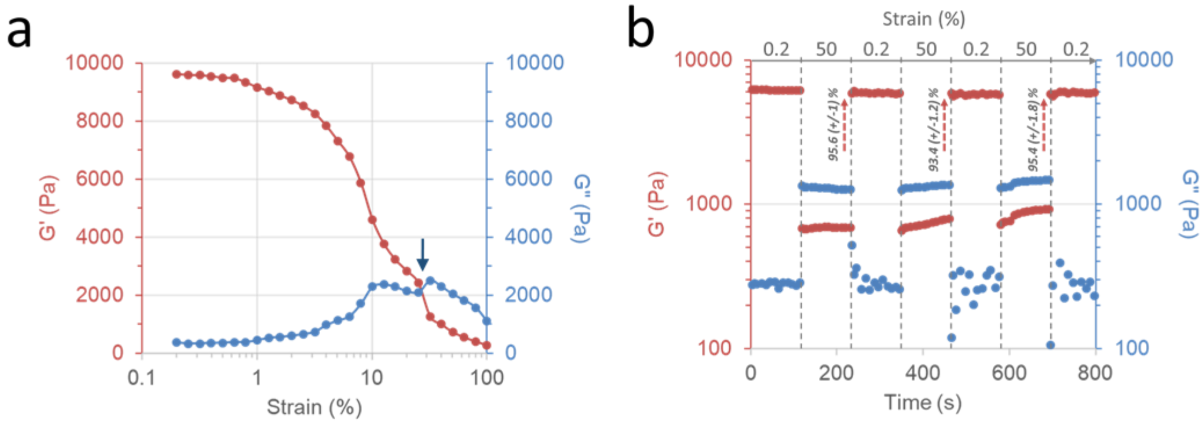

Supplementary figure 1. HA-BP•Laponite gels self-heal following shear. An oscillation sweep of HA-BP•clay hydrogels revealed that increasing the strain from 0.2% to 100% was accompanied by a decrease of storage modulus (G′, red) and an increase of loss modulus (G″, blue) and a crossover point (arrow) presenting a gel-to-liquid transformation at 30% strain value (a). When the strain was returned back from 100% to 0.2% following the collapse of the gel the mechanical properties of the gel were rapidly recovered. A cyclic strain test with strains alternating from low (0.2%) to high (50%) values confirmed the self-healing ability of the hydrogel. Self-healing efficiency under these conditions (labelled up arrows, calculated as percentage first-cycle storage modulus at 0.2 % strain) averaged 95% (+/-1.2%) (b).

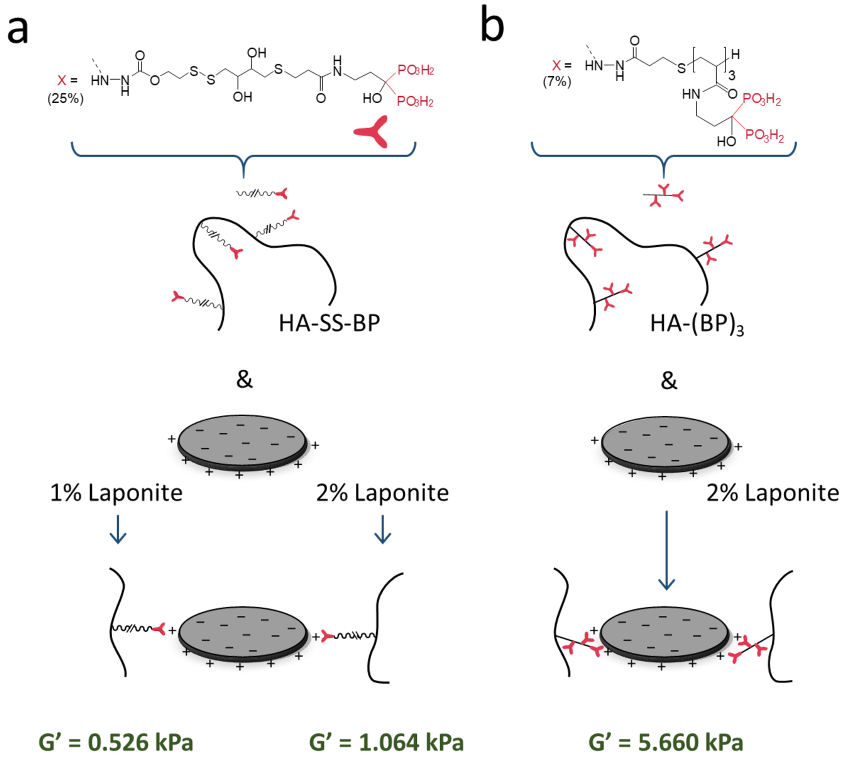

Supplementary figure 2. HA-BP•Laponite physical gel storage modulus was dependent on nanoclay concentration and bisphosphonate attachment chemistry. Increasing the Laponite concentration from 1% to 2% doubled the storage modulus (a). Increasing the number of bisphosphonate groups via an acrylamide-thiol linkage increased stiffness compared with the single and more labile disulfide BP attachment (b). Storage modulus (G') is recorded at 1 Hz.

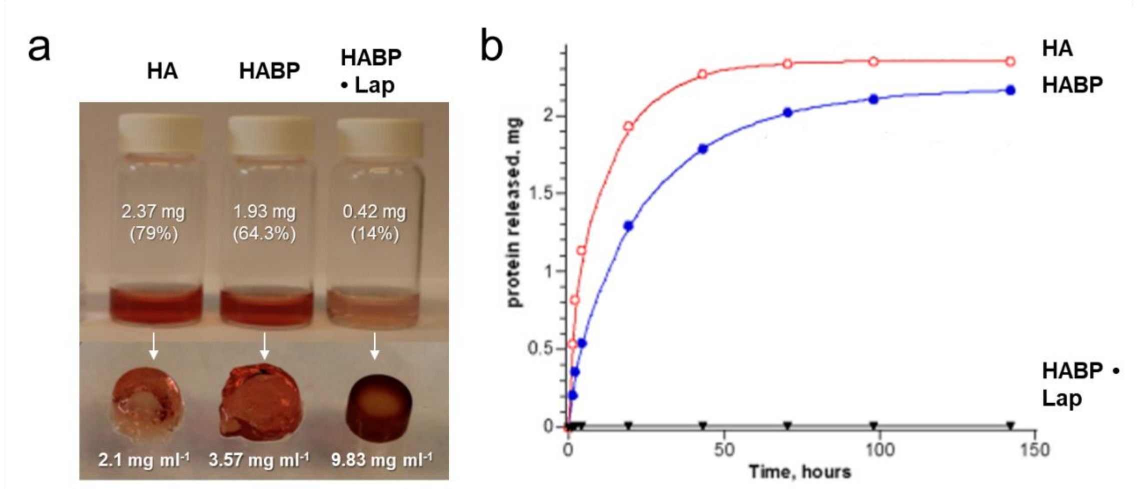

Supplementary figure 3. HA-BP•Laponite physical gel protein loading capacity and release. Following equilibration in a 10 mg ml-1 solution of cytochrome C (Mw 11.7 kDa; PI, 9.6) HA-BP•Laponite gels displayed a very high loading capacity (9.83 mg ml-1) compared with HA and HABP hydrazone cross-linked analogues in the absence of clay (1.08 and 3.57 mg ml-1 respectively) (a). Negligible release of the loaded protein was observed from the HA-BP•Laponite gels over the subsequent 6 day incubation period (b).

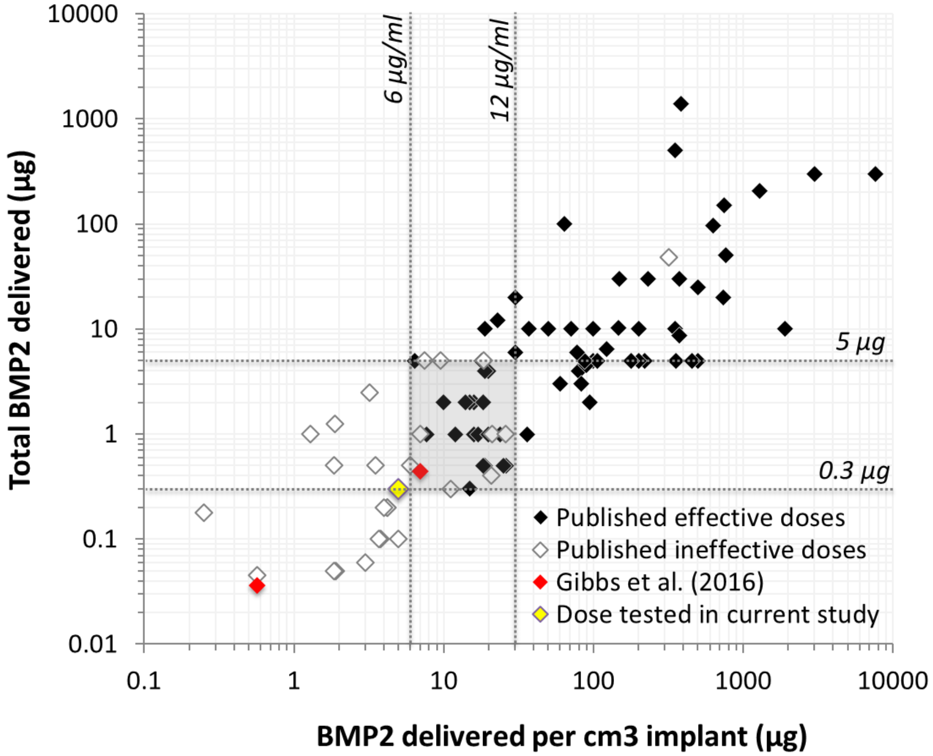

Supplementary figure 4. HABP-Laponite hydrogels sustain robust ectopic bone induction at BMP2 doses below the typical efficacy threshold for BMP2 carriers. The graph plots effective and ineffective total doses against dose per cm^3 implant derived from 72 identified studies testing various carrier materials for BMP2 induction of ectopic bone (subcutaneous or muscle) in small animal models. The shaded region highlights the typical range for biomaterial dose reduction. In previous work (Gibbs et al. 2016) on Laponite colloidal gels ectopic bone induction was detected at total doses of 500 ng and 40 ng, here robust ectopic bone formation was achieved at a dose of 300 ng BMP2 providing evidence that the use of Laponite edge sites for cross-linking does not impede its utility for growth factor delivery. Adapted from [7].

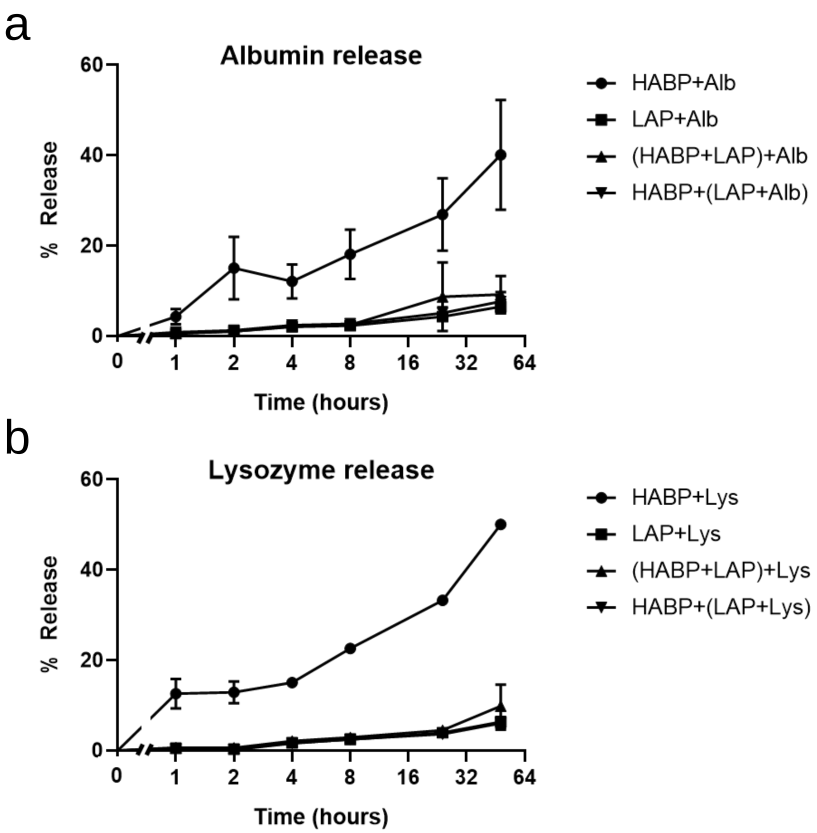

Supplementary figure 5. Protein release from HA-BP•Laponite physical gels is retarded by Laponite and independent of the order of mixing. In contrast to HABP gels alone which showed fast release of both albumin (a) and lysozyme (b), HA-BP•Laponite physical gels, like Laponite alone, displayed minimal release of either protein. Release of each protein was retarded irrespective of whether the protein solution was combined with HABP (-▲-) or Laponite solutions (-▼-) prior to mixing and physical gelation. Error bars = standard deviation (n=3)

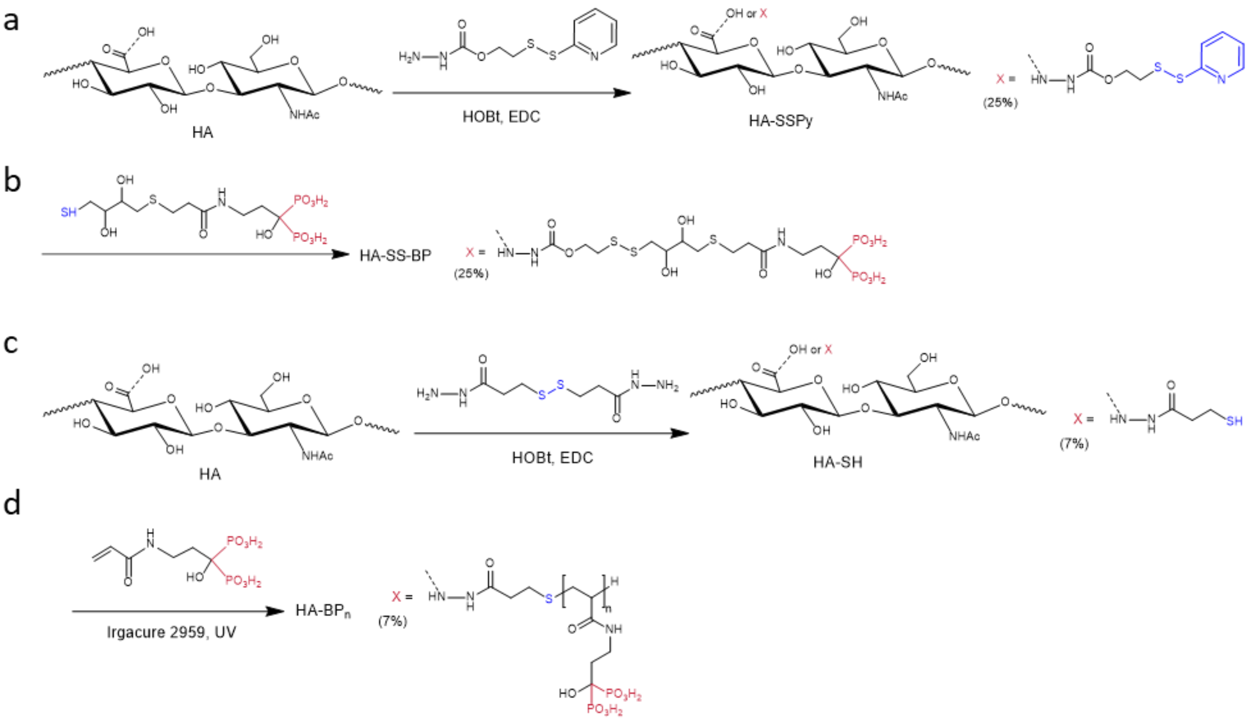

Supplementary figure 6. Preparation of HA-SS-BP and HA-BP_n derivatives. For preparation of HA-SS-BP derivatives, hyaluronan (HA) was modified with 2-dithiopyridyl groups to give HA-SSPy (a) before a thiol-disulfide exchange reaction between HA-SSPy and thiol-derivatized bisphosphonate (b). For preparation of HA-BP_n derivatives, thiol-modified HA was obtained as in (c) and HA-BP_n was obtained by a thiol-ene radical addition reaction between HA-SH and acrylamimde-derivatized bisphosphonate (d).
